# Scalable and cost-effective methods for xenomonitoring of *P. falciparum* and antimalarial drug resistance validated with laboratory and wild-caught mosquitoes

**DOI:** 10.1038/s41598-025-20554-0

**Published:** 2025-09-23

**Authors:** Dario Anvari, Janvier Bandibabone, Andreas A. Kudom, Andreas Wieser, Esto Bahizire, Frank P. Mockenhaupt, Welmoed van Loon

**Affiliations:** 1 Institute of International Health, Charité Center for Global Health, Charité – Universitaetsmedizin Berlin, Berlin, Germany; 2 Laboratoire d’Entomologie Médicale et Parasitologie, Centre de Recherche en Sciences Naturelles (CRSN/Lwiro), Bukavu, Democratic Republic of the Congo; 3 Department of Conservation Biology and Entomology, University of Cape Coast, Cape Coast, Ghana; 4 Institute of Infectious Diseases and Tropical Medicine, University Hospital, Ludwig-Maximilians University Munich, Munich, Germany; 5 Max Von Pettenkofer Institute (Medical Microbiology), Ludwig-Maximilians University Munich, Munich, Germany; 6 Immunology, Infection and Pandemic Research IIP, Fraunhofer ITMP, Munich, Germany; 7 German Center for Infection Research (DZIF), Partner Site Munich, Munich, Germany; 8 Center for Tropical Diseases and Global Health, Catholic University of Bukavu, Bukavu, DR Congo; 9 Department of Nutrition and Health, Centre de Recherche en Sciences Naturelles (CRSN/Lwiro), Bukavu, Democratic Republic of the Congo

**Keywords:** Antimalarial drug resistance, Molecular surveillance, Xenosurveillance, Molecular biology, Malaria, Epidemiology

## Abstract

Human blood samples serve as the gold standard for molecular surveillance of the malaria parasite *Plasmodium falciparum*. However, these samples may not accurately reflect the parasite population and come with logistical constraints and ethical requirements. Using blood-fed mosquitoes as a sample basis could overcome these challenges. We developed and validated DNA extraction methods and PCR assays, allowing for *P. falciparum* detection from whole mosquitoes and sequencing of drug resistance genes. PCRs were consistently positive on mosquito samples mimicking field conditions, i.e., with low *P. falciparum* infection (2000× dilution of a single oocyst-infected mosquito) or after a blood-meal with low parasitemia (250× dilution of 1% parasite density). Antimalarial drug resistance genes *PfK13* and *PfMDR1* could be sequenced from these samples, and markers of resistance could be detected in samples reflecting a mutated minority parasite population of ≥ 25%. Pools of 20 mosquitoes allowed for the detection of a single infected mosquito. We applied our protocol on 50 field-caught *Anopheles* mosquitoes from DR Congo. Five out of eight mosquito pools were *P. falciparum* positive, providing good sequencing reads for *PfK13* and *PfMDR1.* Our presented method has the potential to facilitate surveillance, e.g., on drug resistance, leveraging the promising properties of xenomonitoring**.**

**Supplementary Information:**

The online version contains supplementary material available at 10.1038/s41598-025-20554-0.

## Introduction

Malaria control faces persistent and new challenges, including mutations in the malaria parasite conferring drug resistance or diagnostic evasion^1^. Molecular techniques, especially PCR methods, have contributed significantly to the understanding of such phenomena and today represent an indispensable tool for the highly sensitive detection and characterization of malaria parasites^2^. Applications range from assessing sub-microscopic infection reservoirs to the detection of molecular variants in single parasites. In that, human blood samples serve as the gold standard for the characterization of intraerythrocytic parasites such as *Plasmodium falciparum*, the most dangerous malaria parasite. For isolating *Plasmodium* DNA from blood, in-house protocols and commercial kits are widely available. However, using human samples for research or surveillance of, e.g., antimalarial resistance markers, comes with ethical requirements and logistical constraints. Moreover, human blood samples may not accurately represent the actual parasite population in a given area. For example, when focusing on malaria patients, a bias may arise towards clinical disease, e.g., as to high parasite density infections and potentially more virulent strains. Using blood-feeding mosquitoes as specimens, so-called *xenomonitoring*, could overcome these limitations and allow for large-scale surveillance. *Plasmodium* DNA can be isolated from (i) sporozoites in the salivary glands of infected female *Anopheles* mosquitoes, *Plasmodium’s* obligate invertebrate host (ii) oocysts in these mosquitoes, and from (iii) the midgut of any anthropophilic mosquito (e.g., *Anopheles spp*, *Culex spp*, *Aedes spp*) after a fresh blood meal on an infected human host. Methods for scalable and cost-effective DNA extraction and PCR on mosquitoes carrying *P. falciparum* are scarce and hardly implemented^3,4^, but they are crucial for successful xenomonitoring. We developed and tested such a method, allowing for *P. falciparum* detection from whole mosquitoes along with PCR for drug resistance-associated genes *multidrug-resistance-1* (*MDR1*) and *Kelch-13* (*K13*) for downstream sequencing. Because our model provides only a limited approximation of field conditions with low-infection mosquitoes, we applied our methods also to 50 wild-caught *Anopheles* from the DR Congo, 2023.

## Results

### DNA extraction methods on uninfected mosquitoes

We assessed total DNA yield when extracting from uninfected *Anopheles* mosquitoes with several methods, applying each method on three full mosquitoes each (Table 1). Mosquito homogenization by plastic stick bashing combined with Chelex-based extraction stood out with a mean DNA yield of 2.04 ng/µL, in a final elution volume of 150 µL (e.g., approx. 300 ng DNA per mosquito).

**Table 1:** Total DNA yield of different homogenization and extraction method combinations on full, uninfected mosquitoes.

	Qiagen kit extraction	Chelex-based extraction
	Mean concentration (ng/µL), [individual values]	Mean concentration (ng/µL), [individual values]
No homogenization	0.04 [0.03, 0.04, 0.05]	0.70 [0.56, 0.78, 0.77]
Homogenization with beads and mixing	0.13 [0.12, 0.12, 0.14]	1.67 [1.02, 1.78, 2.22]
Homogenization with stick bashing	0.22 [0.16, 0.17, 0.34]	2.03 [1.79, 1.86, 2.46]

### Limit of detection on samples mimicking a wild-caught Anopheles mosquito

**Fig. 1:**
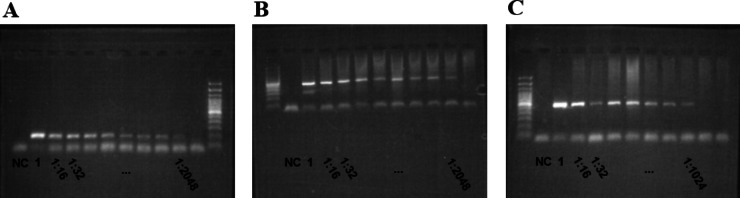
Limit of detection for three PCR assays. Examples of gel electrophoresis runs for 1:2 serial dilutions of sample material mimicking a single oocyst-infected. NC, negative control. *Anopheles* mosquito for *P. falciparum 18s rRNA* (**a**), *K13* (**b**), and *MDR1* (**c**).

*Anopheles coluzzi* mosquitoes were infected by membrane feeding with *P. falciparum* gametocytes of the NF54 strain. The mosquitoes were harvested 11 days post infection, and a random sample of 30 was assessed for oocysts by microscopy of the midgut. Among these, 24 (80%) were infected and the median (interquartile range, IQR) oocyst count was 15 (1.3–34.5). When excluding midguts with zero observed oocysts, these figures were 20 (9–41). We used infected mosquitoes from the same batch to mimic *P. falciparum* infected field mosquitoes by diluting DNA from laboratory infected mosquitoes with high oocyst counts using DNA from uninfected mosquitoes. To assure an oocyst count close to the laboratory batch median, we mixed DNA from three *P. falciparum* positive laboratory infected mosquitoes as starting material. Of note, the mechanical and enzymatic DNA release from ripe oocysts of high abundance from the laboratory infected mosquitoes is likely more successful than single oocyst DNA release from field mosquitoes. In the samples mimicking a single-oocyst infection, *P. falciparum 18s rRNA*, *K13*, and *MDR1* PCRs were positive for dilutions of up to 1:2,000, 1:1,000, and 1:2,000, respectively (Fig. 1).

### Limit of detection on samples mimicking a mosquito after an infectious blood-meal

Samples mimicking a wild-caught mosquito (of any anthropophilic species) directly after a 1% parasitemia blood-meal were prepared by mixing 1.5 µL of *P. falciparum* erythrocyte culture with uninfected mosquitoes followed by DNA extraction. Here also, the release of *P. falciparum* DNA from blood added to the sample outside of the mosquito is likely to be more successful compared to release of DNA from blood in the mosquito stomach. These sample preparations were positive for dilutions of up to 1:250, 1:60, and 1:250.

### Limit of detection on pooled mosquitoes

In the third approach, pools of up to 20 mixed species mosquitoes consistently allowed for the detection of a single laboratory-infected *Anopheles* mosquito (*i.e.*, with expected high oocyst count), as well as for the detection of a prepared sample mimicking a single oocyst-infected *Anopheles* mosquito.

### Sequencing of *P. falciparum* genes and variant detection

**Fig. 2:**
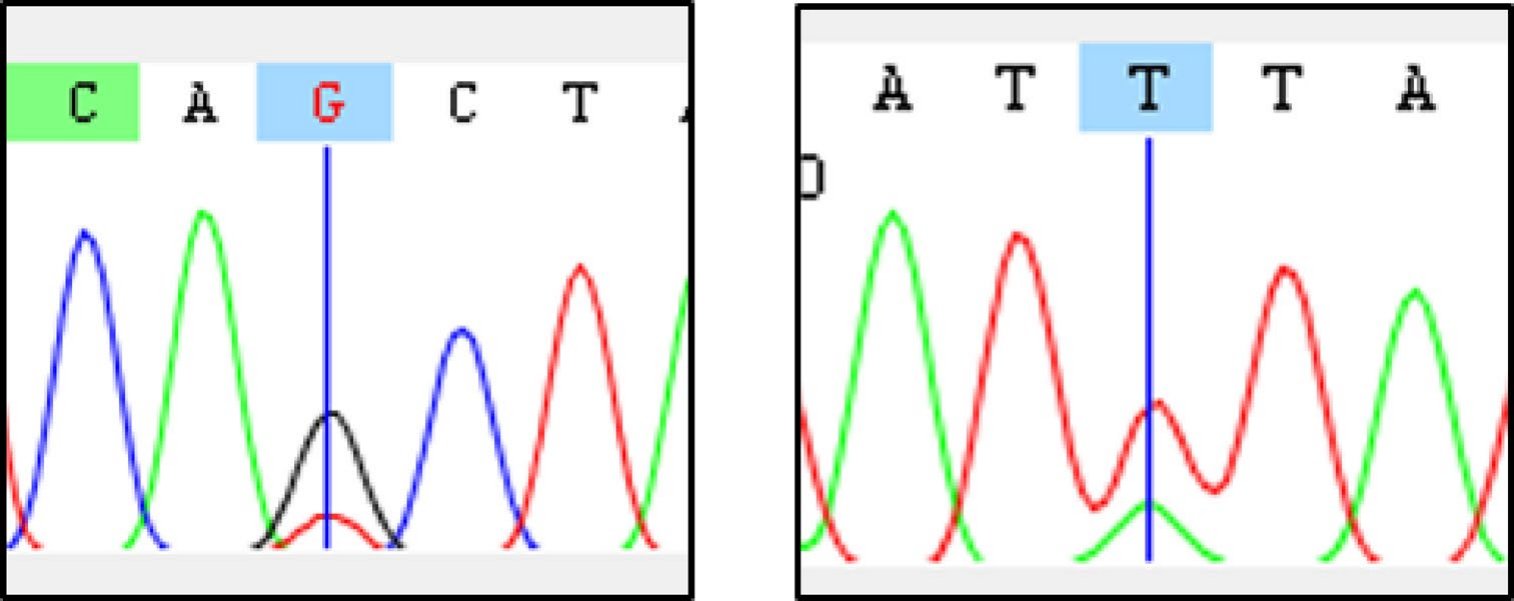
Sanger sequencing chromatogram fragments for *PfK13* and *PfMDR1* of a sample mimicking a mosquito after a blood meal containing 1% parasitemia with 1:4 minority allele *PfK13*-A578S (left) and *PfMDR1*-N86Y (right).

For all mosquito preparations, Sanger sequencing of *PfK13* and *PfMDR1* was successful at any serial dilution with visible bands by gel electrophoresis. Also, in the one preparation including two parasite strains (blood meal approach), minority single nucleotide variants, such as *PfMDR1*-N86Y, could be detected in a ratio of up to 1:3 (Fig. 2).

### Molecular surveillance on caught Anopheles from DR Congo

Lastly, we applied our methods to pooled, wild-caught female *Anopheles* from DR Congo. Five out of eight pools were positive by *P. falciparum 18s rRNA* PCR (2 pools of 10 mosquitoes, 3 pools of 5 mosquitoes). *Plasmodium falciparum* prevalence among single mosquitoes was estimated from the pool positivity by a statistical model using the R-package “PoolTestR” to be 16.0% (95% confidence interval, 5.8–33.4%). All five positive pools were subjected to PCR for *PfMDR1* and *PfK13* and subsequent Sanger sequencing. All pools generated good-quality amplicons and sequencing reads for *PfMDR1*, whereas this was true for four out of five pools for *PfK13*. As for *PfMDR1*, sequence analysis did not reveal traces of the N86Y mutation in any of the pools. In four out of five pools, we found the Y184F mutation (three homozygous, one heterozygous signal). For *PfK13*, sequence analysis revealed two mutations: V650A and A486T, each present in one of the four pools, and each as heterozygous signal in the chromatogram as a minority peak.

## Discussion

The latest world malaria report by WHO names four biological threats to malaria interventions, two of which concern *P. falciparum* directly^1^. One is the spread of mutations conferring antimalarial drug resistance, particularly against artemisinins in East Africa, and the other is the spread of mutations in the histidine-rich protein (HRP) 2 and 3 genes, which render standard HRP-based rapid tests falsely negative. Simple, fast and cost-effective molecular techniques applied on a large scale are essential to assess the extent of only these two threats. Traditional surveys based on blood samples from patients or volunteers are time-consuming, costly, and complex: they require ethical clearance, logistical preparation, trained medical personnel, recruitment processes, provision of medical care, cooperation with often overburdened health systems, and they may only capture subgroups of the parasites to be studied. Molecular surveillance of *Plasmodium* based on mosquitoes could overcome many of these issues. Prerequisites for implementation of xenomonitoring for antimalarial drug resistance are sufficient sensitivity and reproducibility.

In the present study, we demonstrate that molecular detection and genotyping of *P. falciparum* is possible on whole mosquitoes with low-cost and scalable methods for DNA extraction and amplification. Both infected and blood-fed mosquitoes can be used and pooled, facilitating surveillance. Applying this approach to 50 field-caught *Anopheles* mosquitoes from DR Congo, we show the feasibility for genotyping drug resistance genes *PfK13* and *PfMDR1*. The presented method provides a starting point to pilot local surveillance, e.g., on drug resistance, leveraging the promising properties of xenomonitoring.

Protocols for molecular typing of *P. falciparum* from mosquitoes are available^3,4^, but those often use costly commercial DNA extraction kits and require dissection of the mosquito midgut or salivary glands, which is timely and needs trained entomologists. Reports including limits of detection are scarce and rather use uninfected mosquitoes spiked with *Plasmodium* DNA instead of actual infected mosquitoes. Moreover, available methods do rarely cater for scale-up, which is a requirement for surveillance. In the present study, we used infected mosquitoes to assess the limit of detection of a combined DNA extraction and PCR assay, accounting for the potential challenges of releasing *Plasmodium* DNA from the mosquito’s interior. We confirmed that our method does not require dissection of mosquitoes to detect *Plasmodium* DNA. The DNA extraction method is cost-efficient at an estimated cost of 1€ per sample (70% Proteinase K, 20% Chelex®-Resin-100, 10% ddH_2_O, pipettips, tubes) and only requires pipetting in a new tube once. A yield of 300 µg after extraction of mosquito tissue is in accordance with other reports^5^.

PCR assays consistently showed high sensitivity for infected *Anopheles* mosquitoes mimicking single oocyst-infection (limit of detection at 2000–1000× dilutions). Sensitivity for uninfected mosquitoes spiked with an infected “blood meal”, was sufficient (limit of detection at 250–60×  dilutions, corresponding with 0.004–0.02% parasitaemia in the blood meal), but considerably lower than for PCR on mosquitoes mimicking single oocyst-infection. The blood meal sample should theoretically contain more *P. falciparum* DNA copies compared to a single-oocyst infected mosquito, as 1.5 µL blood with 1% parasite density contains roughly 60,000 parasite genome copies (assuming 4,000,000 RBCs/µL blood) *versus* up to 15,000 copies from sporozoites per oocyst found in lab infected mosquitoes^6^. These counterintuitive results may be explained by PCR inhibitory effects of hemoglobin remnants after extraction^7^. Chelex-based extraction does not result in DNA as pure as after silica-based extraction^8,9^. Inhibiting agents might still be present in the end product and therefore adding Proteinase K and a heating step to 99 °C are essential to degrade potentially problematic proteins in the sample. Of note, adding BSA to the PCR assays is crucial to further limit the effect of potential PCR inhibitors. Silica-based DNA extraction requires the use of commercial spin-columns, which are costly, and result in lower amounts of DNA in the end product^9,10^. We have therefore favored the use of our in-house Chelex-based extraction protocol^7^.

The PCR assay for *P. falciparum 18s rRNA* had a limit of detection twice as low as that for *PfK13*. This may be due to the *Pf 18s rRNA* gene being present at 5 copies/genome^11^, whereas *PfK13* occurs only once. However, the *PfMDR1* limit of detection being similar to *Pf 18s rRNA* suggests that the *PfK13* PCR has a sequence-specific sensitivity limitation. Our results indicate that for the sensitive detection of *P. falciparum* DNA in mosquito pools, the *Pf 18s rRNA* PCR should not be replaced by directly using *PfK13* PCR but could be by *PfMDR1* PCR.

Our sequencing results of blood samples with parasites carrying different genotypes indicate that the *PfK13* and *PfMDR1* PCR in combination with Sanger sequencing can detect a mutant parasite clone in a blood meal in a ratio of 1 mutant to 3 wild type single mutations. Mosquitoes may have more than one blood meal from infected hosts^12,13^, and hosts may carry multiple *P. falciparum* strains with distinct genetic structure. For surveillance of molecular markers in *P. falciparum*, we propose a mosquito pool size that corresponds to the probability of containing a maximum of one infected mosquito. Also, a “diagnostic” *Pf 18s rRNA* PCR should first be done on an initial set of individual mosquitoes to estimate *P. falciparum* positivity before pooling.

Due to digestion, human DNA is detectable for only 12 h following a blood meal in *Culex*, up to 48 h in *Aedes*, and up to 72 h in *Anopheles* mosquitoes^14–16^. This suggests that *Plasmodium* DNA in these mosquito species may also only be detectable within this limited time window. On the other hand, (mosquito pools containing) *Anopheles* mosquitoes may carry *Plasmodium* DNA for up to 20 days, after the last oocyst matures^17^, and most *Anopheles* mosquitoes die before then^18^. Thus, *P. falciparum* genotyping results from mixed mosquito pools can theoretically reflect parasite signatures in human hosts from the time of infection for up to 3 weeks after.

We applied our methods on field-caught *Anopheles* mosquitoes from the high malaria transmission area of Kibali in DR Congo^19^. Our PCR results revealed that almost 20% of the *Anopheles* carried *P. falciparum* DNA. The mutations we found in *PfK13*, a gene associated with partial artemisinin resistance which recently emerged in East Africa^20^, are of unknown relevance. *Plasmodium falciparum MDR1* mutations at loci 86 and 184 are linked to sensitivity to several antimalarial drugs^21^, and our observed pattern matches previous observations from the region^22,23^. These results merely serve as a demonstration of feasibility. One positive pool failed to amplify *PfK13*. Potentially, storage conditions of these mosquitoes, i.e., at ambient temperature and on silica gel, affected DNA quality, compared to the infected mosquitoes we used for PCR validation that were refrigerated and stored in ethanol. However, drying of mosquitoes is considered to not lead to loss of sensitivity^24,25^. The unexpected negative *PfK13* PCR highlights the need to validate the assays extensively on wild-caught mosquitoes.

A limitation of our study is that the laboratory-infected mosquitoes carried much more oocysts in their midgut (a median of 20) compared to infected field mosquitoes for which reported averages range from 2 to 10 oocysts^26–28^. We diluted the laboratory-infected mosquito DNA with uninfected mosquito DNA to mimic a single-oocyst infection, but this may not have sufficiently represented the low probability of releasing a very low parasite load from mosquito tissue. This holds also true for samples mimicking mosquitoes after an infectious blood meal. Our molecular methods should be validated and further assessed on a model better representing low-infection scenarios and the distribution of all parasite stages. E.g., by setting up and validating *Anopheles* feeding assays resulting in a low and consistent infection load, by harvesting the mosquitoes at 8, 10, 13, and 15 days post infection, by using *P. falciparum* field isolates with different drug resistance signatures for the feeding assays, and by assessing other mosquito species after blood feeding with these parasites.

Today’s malaria surveillance is unthinkable without molecular techniques. We present a sensitive and scalable method for molecular surveillance of *Plasmodium* using anthropophilic mosquitoes, for example to screen for antimalarial resistance markers. Xenomonitoring has a clear potential for up-scaling, and may overcome patient selection bias. With growing molecular biology capacities in Sub-Saharan Africa, opportunities arise for local, fast, cheap and sustainable surveillance using mosquitoes. Implementation should be tailored to local capacities and evaluated for usability and for sensitivity on wild-caught mosquitoes.

## Methods

For method development and evaluation, we used various sources and types of mosquitoes. Uninfected *Anopheles spp*, *Aedes spp*, and *Culex spp* mosquitoes were reared from field-caught larvae in Ghana and Ethiopia. These mosquitoes were killed as adults, dried, shipped at ambient temperature and stored at − 20 °C without further preservation. Laboratory infected *Anopheles coluzzii* mosquitoes (Ngousso S1) were reared in Berlin at 28 °C and 70–80% humidity with a 12/12 day/night cycle. Mosquitoes were 4–5 days old at the time of infection. Infection was done by 15 min of feeding on a membrane feeder with stage V gametocytes (approximately 4.9 × 10^6^ gametocytes/ml, in human serum with fresh erythrocytes at 50% hematocrit). Unfed mosquitoes were removed from the infection batch. Infected mosquitoes were killed in ethanol 70% at 11 days post infection, rinsed in PBS and kept at 4–8 °C. A random selection of 30 mosquitoes was decapitated and their midguts dissected. These were stained for 10 min with 1% mercurochrome in water in a humidified chamber. Oocysts were counted for each midgut under a light microscope by a trained microscopist. 80% of assessed midguts contained oocysts, and among these, the median oocyst count was 20 (IQR, 9–41).

In short, we assessed DNA extraction methods from three uninfected *Anopheles* mosquitoes, each, using three homogenization approaches (1 a-c) and a commercial *versus* an in-house DNA extraction method (2 a,b). DNA yield was measured (3), and the method with the highest DNA yield was then further used to optimize existing PCR assays for *P. falciparum* targeting *18s rRNA*, *K13*, and *MDR1* on infected mosquitoes (4 a-c). Limits of detection for each PCR assay were assessed by serial dilutions of different samples including laboratory-infected *Anopheles* mosquitoes and reared *Anopheles*, *Aedes*, and *Culex* mosquitoes (5 a-c). Finally, PCR amplicons were Sanger sequenced and aligned in CodonCode Aligner Version 9.0.1 to respective reference genes from PlasmoDB (www.plasmodb.org). In detail:

1. *Sample preparation and homogenization*: single, uninfected *Anopheles* mosquitoes were put in a 1.5 mL microcentrifuge tube, submersed in 20 µL PCR-grade double deionized H_2_O (ddH_2_O) and subjected to homogenization by (a) plastic beads (approx. 8–10 beads per tube, size 1 mm), vortexing and centrifugation at 10,000×*g* for 10 min, (b) manual bashing with a sterile plastic stick (diameter 3 mm, e.g., an inoculation bacteriology loop) until a homogenous mass, or (c) no mechanical homogenization. Each approach was repeated on six mosquitoes, which were then randomly divided in two batches for testing DNA extraction methods.
2. *DNA extraction* was done by either (a) QIAmp DNA Mini Kit (Qiagen), following the manufacturer’s protocol for tissue lysis, or by (b) an in-house protocol using Chelex. For the Chelex protocol, 20 µL proteinase K (800 units/ml) was added to each sample and incubated for 5 h at 37° C while gently mixing. After spinning down, 70 µL ddH_2_0 and 100 µL Chelex®-Resin-100 (mesh 50–100) 40% w/v were added. We punched a hole in each microcentrifuge cap with a sterile, hollow syringe needle to allow for pressure release, and heated the samples to 99 °C for 10 min while gently mixing. Samples were centrifuged at 20,000×*g* for 10 min and the supernatant containing the DNA-extract was carefully pipetted into a new 1.5 mL microcentrifuge tube while avoiding carry-over of any Chelex beads (final volume approx. 150 µL).
3. After extraction, DNA quantity of each sample was measured by a Quantus fluorometer using the QuantiFluor dsDNA system (i.e., double-strand DNA binding Dye and Lambda DNA Standard, Promega).
4. *PCR assays* for (a) *Pf 18s rRNA*, (b) *PfK13,* and (c) *PfMDR1* were optimized for each homogenization-extraction combination. To each assay, we added Bovine Serum Albumin (BSA) to inhibit potential PCR-inhibitors (e.g., hemoglobin), and additional MgCl_2_ to compensate for the chelating effect of BSA. PCR outcome was assessed by electrophoresis of 5 µL PCR-product on a 1% agarose gel with ethidium bromide, and UV-transillumination. The final PCR assays contained, in a 20 µL reaction volume, 0.2 mM dNTPs (each, New England Biolabs), 1× B1 solution (Solis Biodyne), 1.5 U hot-start taq polymerase (5 U/µL HOT FIREpol, Solis Biodyne), 10, 2.5, and 5 mM MgCl_2_ for *Pf 18s rRNA*, *K13*, and *MDR1*, respectively, and 0.3 µM forward and reverse primers (all from Eurofins Genomics; Germany; *Pf 18s rRNA*, forward, 5′-TTA AAC TGG TTT GGG AAA ACC AAA TAT ATT -3′, reverse, 5′-ACA CAA TGA ACT CAA TCA TGA CTA CCC GTC-3′^29^; *PfK13*, forward, 5′- GGG AAT CTG GTG GTA ACA GC-3′, reverse, 5′-GCC AAG CTG CCA TTC ATT TG-3′^30^; *PfMDR1*, forward, 5′-TTA AAT GTT TAC CTG CAC AAC ATA GAA AAT T-3′^31^). Thermocycling conditions for each PCR-assay were: 95 °C for 15 min; 40 cycles of 94 °C for 1 min, annealing temperarute for 1 min, 72 °C for 1 min; and final elongation at 72 °C for 10 min. Assay-specific annealing temperature *Ta* was 58 °C for *Pf 18s rRNA*, 61 °C for *PfK13*, and 62 °C for *PfMDR1*.
5. *Limits of detection* were assessed for each PCR-assay by 1:2 serial dilutions on different sample types, each done on three replicated sample preparations. These sample types were designed to mimic the following field conditions: (a) one *Anopheles* mosquito with single oocyst-infection; (b) one uninfected mosquito after an infected blood meal with various multiplicity of infection; (c) pooled mosquito batches containing a single infected *Anopheles* mosquito. The details of these *Anopheles* preparations were as follows: (a) *single oocyst-infected Anopheles:* From the laboratory *Anopheles* infection batch (median oocyst count, 20 per infected midgut; IQR, 9–41), DNA extracts of three laboratory-infected *Anopheles* mosquitoes were randomly selected and PCR-confirmed for *P. falciparum*. These were mixed together to control for infection load variations between individual mosquitoes, then 1:20 diluted with uninfected mosquito DNA; (b) *mosquito after blood-meal:* Uninfected *Anopheles* mosquitoes were spiked with 1.5 µL infected blood with 1% parasite density from *P. falciparum* erythrocyte cultures. We used two *P. falciparum* clones with distinct *PfK13* and *PfMDR1* genotypes for this: either the wild type clone was used, or the two culture clones were mixed in different ratios to represent different infection clonality. Mixing ratios were 1:1, 1:2, 1:3; (c1) *mosquito pools:* pools of 4, 9, or 19 uninfected *Aedes, Culex,* and *Anopheles* mosquitoes with one laboratory-infected *Anopheles* mosquito, in triplicate; and (c2) pools of 5, 10, or 20 uninfected *Aedes*, *Culex,* and *Anopheles* mosquitoes spiked with 1 µL DNA from 5 a) (the sample preparation mimicking an *Anopheles* mosquito with single oocyst-infection) to represent the DNA load of a pooled field-caught mosquito, in triplicate. We reported limits of detection if they were consistent for at least three replicates of each sample type on consecutive occasions. Uninfected mosquito DNA used for dilutions was separately tested for *P. falciparum* DNA by PCR to confirm negativity. Positive and negative controls were included in each independent PCR run, consisting of extracted DNA from NF54 and ddH_2_O, respectively.

Eventually, we applied the three PCR assays on 50 wild-caught *Anopheles* mosquitoes from the Kibali gold-mine area, DR Congo, 2023. These *Anopheles* were shipped at ambient temperature and pooled (2 pools containing 10 mosquitoes each, and 6 pools containing 5 mosquitoes each). To estimate individual *P. falciparum* prevalence from these pooled samples, we assumed 100% sensitivity and specificity of the PCR detection method. We applied a Bayesian framework to estimate prevalence, using the “PoolPrev” function from R Package “PoolTestR”^32^ in R version 4.3.1. Function parameters used were: the default uninformative “Jeffreys” prior as prior belief for the prevalence, “reproduce.poolscreen” set to FALSE, the defaults for Markov chain Monte Carlo and control options (2000 iterations, warmup 1000, 4 chains, delta 0.9). The prevalence reported was a maximum likelihood estimate with respective 95% confidence interval.

## Supplementary Information

Below is the link to the electronic supplementary material.

Supplementary Material 1

## Data Availability

Sequencing data generated or analyzed during this study are included in this published article as supplementary files.
